# Cellular Senescence in Health, Disease and Aging: Blessing or Curse?

**DOI:** 10.3390/life11060541

**Published:** 2021-06-09

**Authors:** Markus Riessland

**Affiliations:** 1 Department of Neurobiology and Behavior, Stony Brook University, Stony Brook, NY 11794, USA; markus.riessland@stonybrook.edu; 2 Center for Nervous System Disorders, Stony Brook University, Stony Brook, NY 11794, USA

Sixty years ago (1961), Hayflick and Moorhead reported that primary cells terminate their growth and stop dividing after ~50 passages or one year in culture. This seminal study described the phenomenon that we now refer to as “cellular senescence” [1]. More specifically, the description by Hayflick and Moorhead unraveled “replicative senescence”, which is caused by cell-division-dependent telomere attrition. Since then, increasing numbers of additional senescence-inducing factors have been identified. In parallel, a plethora of cell types have been recognized to possess the ability to enter a state of cellular senescence. These studies revealed diverse senescence-related cellular phenotypes and identified various metabolic changes, gene-activity alterations and other molecular markers [2,3,4]. Although some gene expression changes are characteristic hallmarks of cellular senescence, a single molecular marker has not been identified. Accordingly, the univocal identification of a senescent cell remains challenging. To address this problem, the International Cell Senescence Association (ICSA) assembled a list of key features observed in senescent cells [2].

A particularly interesting feature of senescent cells is the so-called senescence-associated secretory phenotype (SASP), which remodels the gene epression profile of a senescent cell causing the secretion of proinflammatory molecules to signal to the immune system “come here and remove me”. During development, and in organisms with fully functional immune systems, senescent cells are usually detected and cleared from the tissue [5]. In case where immune cells do not remove the senescent cells, they remain in the tissue and continue to express the SASP. In turn, this would cause a damaging local inflammation and could also induce remodeling of the surrounding tissue as well as the spreading of senescence. Aged organisms possess a significantly reduced regenerative potential and immune function resulting in the accumulation of senescent cells [5]. Interestingly, this accumulation has also been observed in age-related disorders, neurodegenerative diseases, cardiovascular diseases, and others [6,7]. Because of its detrimental effect on the surrounding tissue, the accumulation of senescent cells is not just a consequence, but can instead be understood as a major driver of aging. Accordingly, recent studies described that the removal of senescent cells showed beneficial effects on healthspan and lifespan [8]. This exciting research led to the discovery of “senolytics”, drugs which can kill senescent cells. Moreover, because of the heterogeneity of cell types that show senescence-like phenotypes, including cardiovascular cells and post-mitotic neuronal cells [6,9,10], further research is required to unravel the molecular background that renders a cell type vulnerable to senescence and to determine the pathways that induce senescence in a cell type-specific manner.

Given that there are many open questions in the field, this Special Issue of *Life* was created to shed light on the molecular pathways of cellular senescence, inflammaging, and the possible strategies to interfere with these processes. The work published in this Special Issue of *Life*, entitled “Cellular Senescence in Health, Disease and Aging: Blessing or Curse?”, mirrors the broad interest in the field of cellular senescence since the presented studies highlight quite diverse aspects of senescence and related pathways from various areas of research. 

The manuscript by Panchanathan et al. reports observations that identify the interferon inducible POP3 PYHIN protein as a potential negative regulator of the AIM2 inflammasome and SASP in senescent human prostate epithelial cells. This study provides insight into the age-related development of prostatic inflammatory diseases [11].

Senescence DNA damage foci (SDF) and telomere-dysfunction-induced foci (TIF) can be identified by the histone marker γH2AX for cellular senescence and DNA damage, respectively, which makes γH2AX a useful tool for the identification of these traits in diverse tissues [12]. In this Special Issue, Siddiqui and colleagues determine the feasibility of using γH2AX as a molecular biomarker of DNA damage in Alzheimer’s disease (AD). The authors report a protocol that employs laser scanning cytometry (LSC) to measure endogenous γH2AX in buccal cell nuclei from mild cognitive impairment (MCI) patients, AD patients, and healthy controls [13].

Secreted protein acidic and rich in cysteine (SPARC), a molecule that has been described to be overexpressed in senescent cells [14], was the topic of an Opinion manuscript by Ghanemi et al. [15]. The authors emphasize that SPARC not only acts as a regeneration factor but also counteracts the aging-related decrease in regeneration ability, and thus can be seen as a potential factor for preventing age-related conditions.

p16^INK4A^, which is often highly upregulated in many types of cellular senescence, acts as a tumor suppressor and is frequently reduced in human cancers. In this Special Issue, Leon et al. review the potential role of p16 in the regulation of immunological surveillance. In brief, the authors discuss the hypothesis that a p16-positive tumor would foster immunosurveillance by inviting immune cells into the tumor microenvironment, whereas a p16-null tumor would reduce immunosurveillance and promote tumor growth [16].

Finally, two reviews from the Orr lab highlight the importance of cellular senescence in the human brain. Gillispie et al. summarize the role of mitotic cells in brain senescence and discuss implications in neurodegenerative diseases and cancer [17]. The second manuscript reviews the recent discovery of post-mitotic senescence in the brain. In short, Sah et al. provide a comprehensive overview of the current knowledge of the cellular senescence of brain cells, including neurons [18]. Additionally, this manuscript gives an elegant introduction into the field of cellular senescence.

Generally, I hope that this Special Issue of *Life* will capture the attention of both specialists and non-specialists who are interested in understanding the molecular processes involved in cellular senescence and inflammaging. As seen in the diverse articles in this Special Issue, cellular senescence and the molecules that are crucial in its underlying pathways are of high interest in many areas of research. The rising interest in a more thorough understanding of cellular senescence is reflected by the fact that the National Institutes of Health (NIH) have recently established the Common Fund’s Cellular Senescence Network (SenNet) Program to identify and characterize the differences in senescent cells within the body, across various states of human health, and throughout lifespan. It is an exciting time for researchers working on senescence and aging, and overall, there is great hope that the outcome of this research can translate into strategies that provide beneficial effects on healthspan and lifespan in humans. 
